# Surgical Outcomes for C_2_ Tear Drop Fractures: Clinical Relevance to Hangman's Fracture and C_2‐3_ Discoligamentous Injury

**DOI:** 10.1111/os.13163

**Published:** 2021-11-17

**Authors:** Sung‐Kyu Kim, John M. Rhee, Eric T. Park, Hyoung‐Yeon Seo

**Affiliations:** ^1^ Department of Orthopaedic Surgery Chonnam National University Medical School and Hospital Gwangju Republic of Korea; ^2^ Department of Orthopaedic Surgery, Emory Spine Center Emory University Atlanta Georgia USA; ^3^ Department of Biology, College of Arts and Sciences Emory University Atlanta Georgia USA

**Keywords:** Axis, Discoligamentous injury, Hangman's fracture, Surgical outcome, Tear drop fracture

## Abstract

**Objective:**

To analyze characteristics of surgically managed tear drop (TD) fractures of the C_2_ axis associated with other injuries such as hangman's fracture and C_2‐3_ discoligamentous injury as well as treatment outcomes.

**Methods:**

A total of 14 patients (eight men and six women) with TD fractures of the C_2_, who were surgically treated at four national trauma centers of tertiary university hospitals from January 2000 to December 2017, were included in this retrospective study. The mean age of the patients was 45.5 years (ranging from 19 to 74 years). The characteristics, surgical treatment methods (anterior fusion *vs* posterior fusion), and results of 14 TD fractures of the C_2_ were analyzed retrospectively. And the clinical relevance between C_2_ TD fracture and hangman's fracture and C_2‐3_ discoligamentous injury was investigated through the co‐occurrence between injuries. The mean follow‐up time after surgery was 22.6 months (ranging from 12 to 60 months).

**Results:**

Among 14 patients with TD fracture of the C_2_, four patients (28.6%) had anterior TD fracture and 10 patients (71.4%) had posterior TD fracture. All 10 posterior TD fracture patients had anterior C_2‐3_ displacement. While two of four anterior TD fracture patients had posterior C_2‐3_ displacement, the remaining two did not. All 14 patients of TD fracture had at least two or more other associated C_2_ injuries as well as C_2‐3_ discoligamentous injuries. About 92.9% (13/14) of the patients had typical or atypical hangman's fracture; 100% (10/10) of the posterior TD fracture patients had hangman's fracture, but 75% (3/4) of the anterior TD fracture had hangman's fracture. At admission, 13 patients were neurologically intact. However, the remaining patient had spinal cord injury with American Spinal Injury Association (ASIA) impairment scale B with C_2‐3_ bilateral facet dislocation. All four anterior TD fracture patients underwent posterior C_2‐3_ fusion. While four of 10 posterior TD fracture patients underwent C_2‐3_ anterior fusion, the remaining six underwent posterior fusion. At last follow‐up, 100% (14/14) of the patients achieved solid fusion, and visual analog scale for neck pain was significantly improved (5.9 *vs* 2.2, *P* < 0.001). One patient with ASIA impairment scale B had significantly improved to scale D. No major complications occurred.

**Conclusion:**

Our study showed that surgically managed TD fractures of the C_2_ showed a high incidence of other associated spine injuries including hangman's fracture and C_2‐3_ discoligamentous injury. Therefore, special attention and careful radiologic evaluation are needed to investigate the presence of other associated spine injuries including hangman's fracture and C_2‐3_ discoligamentous injury, which are likely to require surgery.

## Graphical abstract

Surgically managed C_2_ TD fractures showed high incidence of associated C_2_ and C_2‐3_ injuries such as hangman's fracture and C_2‐3_ discoligamentous injury, but satisfactory treatment outcomes were obtained. Sagittal computed tomography scan and magnetic resonance imaging (A‐D, B‐E, C‐F) showing tear drop fracture, C_2‐3_ slip, and C_2‐3_ discoligamentous injury.

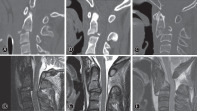

## Introduction

Tear drop (TD) fractures of the C_2_ are rare traumatic injuries of the upper cervical spine, representing about 9%–12% of upper cervical spine injuries and 1%–3% of all cervical spine injuries^1^, ^2^, ^3^, ^4^, ^5^, ^6^. Tear drop fractures of the C_2_ axis differ in several ways from TD fractures in the lower cervical spine. In general, TD fractures of the cervical spine are classified into extension TD fractures caused by hyperextension and flexion TD fractures caused by flexion‐compression force based on injury mechanism by Allen's classification of lower cervical spine injury^1^, ^2^, ^3^, ^4^, ^7^, ^8^, ^9^. Flexion TD fractures commonly occur at the C_4_–C_7_ vertebra and extension TD fractures occur more commonly at C_2_ or C_3_.

Both extension and flexion TD fractures of the lower cervical spine are anatomically anterior TD fractures. Like anterior TD fractures of the lower cervical spine, TD fractures of the C_2_ reported in previous studies are extension TD fractures in terms of injury mechanism and anterior TD fractures anatomically. However, due to the specific anatomical and biomechanical characteristics of the C_2_ vertebra, TD fractures of C_2_ can occur as posterior TD fractures as well as anterior TD fractures depending on complex injury forces. In our treatment experience, C_2_ TD fractures that required surgery were more common in posterior TD fractures than in anterior TD fractures.

To date, a few studies, including case reports or small number of case series, have reported treatment methods and outcomes for TD fracture of the C_2_ ^1^, ^2^, ^3^, ^4^. Tear drop fractures of C_2_ can occur alone or in conjunction with other associated spine injuries, especially C_2‐3_ and other C_2_ injuries. In general, C_2_ fractures heal well with conservative treatment. In a review of the literature, the success rate of conservative treatment for C_2_ fractures was 78.4%^10^, ^11^. Like other C_2_ fractures, most C_2_ TD fractures are successfully managed with conservative measures^7^, ^8^. However, close analysis of previous studies showed that the C_2_ TD fractures successfully managed with conservative treatment were simple or small‐sized TD fractures and did not include complex TD fractures of the C_2_ with other associated injuries. A few previous studies, mostly case reports, have reported that huge or large‐sized TD fractures of C_2_ need to be treated surgically.

In many cases of C_2_ TD fracture requiring surgery, we could observe that it accompanies hangman's fracture. Since associated C_2_ and C_2‐3_ injuries have a significant effect on treatment methods and outcomes, it is important to ensure that comprehensive injury analyses are performed to obtain satisfactory treatment outcomes. However, the criteria for determining the indications for surgery and the best surgical approaches needed to treat C_2_ TD fractures have not been established and are controversial. Little information is available regarding C_2_ TD fracture, such as C_2‐3_ injury pattern, C_2‐3_ discoligamentous injury, other associated cervical spine injury, and neurologic status, which affect treatment methods and outcomes. Additionally, all TD fractures of the C_2_ described in previous studies were anterior, and there are no reports on posterior TD fractures^1^, ^2^, ^3^, ^4^, ^7^, ^8^, ^12^, ^13^, ^14^, ^15^, ^16^, ^17^.

Therefore, the purpose of this study was to investigate the characteristics, relevance to other injuries such as hangman's fracture and C_2‐3_ discoligamentous injury, and surgical outcomes of surgically managed TD fractures of the C_2_.

## Materials and Methods

### Inclusion and Exclusion Criteria

A total of 60 patients with anterior and posterior TD fractures of the C_2_ body were identified from the database of four national trauma centers of tertiary university hospitals between 1 January 2000 and 31 December 2017. The inclusion criteria of this study were as follows: (i) patients with acute trauma history; (ii) patients with TD fracture of the C_2_ as diagnosed on lateral X‐ray and sagittal computed tomography (CT); (iii) patients who underwent surgical treatment for C_2_ TD fractures; and (iv) patients with a minimum follow‐up of 12 months after surgery. Exclusion criteria were as follows: (i) patients with a history of previous surgery or fracture; (ii) patients with a history of rheumatoid arthritis such as ankylosing spondylitis; and (iii) patients with cases of pathologic fracture such as infection and tumor.

### Stratification of Tear Drop Fracture

Plain radiographs, CT scans including sagittal CT, magnetic resonance imaging results, and medical records were retrospectively reviewed. All 14 patients were stratified into anterior or posterior TD fracture of the C_2_ based on location on lateral X‐ray and sagittal CT. Anterior TD fracture of the C_2_ was defined as a fracture that involved the anteroinferior portion of the C_2_ body. Posterior TD fracture of the C_2_ was defined as a fracture that involved the posteroinferior portion of the C_2_ body.

### Radiological Assessment of Characteristics of C_2_ Tear Drop Fractures

#### Direction of C_2‐3_ Displacement

Anterior C_2‐3_ displacement was defined as the case where anterior cortex of the C_2_ body was displaced forward than that of C_3_ body. Posterior C_2‐3_ displacement was defined as the case where anterior cortex of the C_2_ body was displaced backward than that of C_3_ body.

#### C_2‐3_ Discoligamentous Injury

The C_2‐3_ discoligamentous injury, including anterior longitudinal ligament (ALL), disc, posterior longitudinal ligament (PLL), was investigated on sagittal magnetic resonance imaging by discontinuity and signal changes.

#### Other Associated Spine Injuries

The presence of other associated C_2_ injuries, including of the pars interarticularis, pedicle, superior articular facet, transverse foramen, lamina, and spinous process, was investigated on plain radiographs and CT scans including sagittal and coronal CT.

#### Surgical Treatment Methods and Assessment of Fusion Status

Surgical treatment methods were investigated including surgical approach, fusion and fixation methods, and bone graft materials. Fusion status was evaluated by flexion and extension lateral radiographs and CT scans including coronal and sagittal CT. The criteria for bone fusion were as follows: (i) difference of segmental motion less than 2° between flexion and extension lateral radiographs, (ii) formation of a bony bridge, and (iii) no findings of fixation failure.

### Clinical Assessment of Characteristics of C_2_ Tear Drop Fractures

#### Neurologic Status by American Spinal Injury Association (ASIA) Impairment Scale

Neurologic status was evaluated using the ASIA impairment scale. The ASIA scale was used to evaluate the neurological function (sensory and motor) affected by spinal cord injury (SCI): Grade A, A complete SCI. There is no motor or sensory function left below the level of injury; Grade B, Some sensory function but no motor function; Grade C, Motor grade less than 3 below the neurologic level of injury; Grade D, Motor grade of at least 3 below the neurologic level of injury; Grade E, Normal motor and sensory examinations^18^.

#### Visual Analog Scale (VAS) for Neck Pain

Neck pain VAS is a continuous scale to measure subjective pain intensity. For pain intensity, the scale is most commonly anchored by “no pain” (score of 0) and “worst imaginable pain” (score of 10). Neck pain VAS was used to evaluate clinical outcome for pain improvement after treatment.

### Statistical Analysis

Statistical analysis was performed using IBM SPSS Statistics for Windows/Macintosh, Version 22.0 (IBM Corp., Armonk, NY, USA). The difference between initial and last follow‐up neck pain VAS was analyzed by paired *t*‐test. A *P* value less than 0.05 was considered significant.

### Ethics Approval

This multicenter retrospective study was approved by the institutional review board (CNUH‐2020‐337) of the corresponding author's university hospital, and informed written consent was waived from the patients for participation in this study and use of accompanying images.

## Results

### Demographic Data

Out of 60 patients, 14 patients met both criteria and were included in this study. Demographic data and information regarding injury mechanism are summarized in Table 1. The mean age at the time of surgery was 45.5 years (ranging from 19 to 74 years). The mean follow‐up after surgery was 22.6 months (ranging from 12 to 60 months). Eight patients were men and six were women. Regarding the injury mechanism, six patients (42.9%) were involved in a traffic accident, six (42.9%) experienced a fall from height, and two (14.3%) slipped.

**TABLE 1:** Demographic data and information of injury mechanism, the direction of C_2‐3_ displacement, C_2‐3_ discoligamentous injury, associated spine injury, and neurologic status.

Variables	Anterior TD Fx	Posterior TD Fx
	(*N* = 4)	(*N* = 10)
Demographic Data Age Sex (Male/Female) Follow‐Up	46.6 years (range, 37–50 years) 3/1 25 months (range, 12–40 months)	45.0 years (range, 19–74 years) 5/5 21.0 months (range, 12–60 months)
Injury Mechanism Fall down Slip down Traffic accident	2 (50%) 1 (25%) 1 (25%)	4 (40%) 1 (10%) 5 (50%)
Direction of C_2‐3_ Displacement Anterior Posterior No	2 (50%) 2 (50%)	10 (100%)
C_2‐3_ Discoligamentous Injury ALL Disc PLL	4 (100%) 4 (100%) 2 (50%)	10 (100%) 10 (100%) 10 (100%)
Associated Spine Injury C_1_ Atlas C_2_ Axis (except TD Fx) Hangman's fracture C_3‐7_ or T/L Spine	1 (25%) 4 (100%) 3 (75%) 2 (50%)	1 (10%) 10 (100%) 10 (100%) 2 (20%)
Neurologic Status Intact ASIA Impairment Scale	3 (75%) B: 1 (25%)	10 (100%)

ALL, anterior longitudinal ligament; ASIA, American Spinal Injury Association; Fx, fracture; PLL, posterior longitudinal ligament; TD indicates tear drop; T/L, thoracolumbar.

### Stratification of Tear Drop Fracture

Among 14 patients with TD fracture of the C_2_, four patients (28.6%) had anterior TD fracture and 10 patients (71.4%) had posterior TD fracture.

### Radiological Results of C_2_ Tear Drop Fractures

#### Direction of C_2‐3_ Displacement

The information of direction of C_2‐3_ displacement, C_2‐3_ discoligamentous injury, and other associated spine injury are summarized in Table 1. While all 10 of those with posterior TD fracture cases (100%) had anterior C_2‐3_ displacement, two out of four anterior TD fracture cases (50%) had posterior C_2‐3_ displacement, but the remaining two anterior TD fracture cases (50%) did not.

#### C_2‐3_ Discoligamentous Injury

All 10 cases of posterior TD fracture with anterior C_2‐3_ displacement (100%) had C_2‐3_ discoligamentous injuries including the ALL, disc, and PLL. However, while two cases of anterior TD fracture with C_2‐3_ posterior displacement showed all three types of C_2‐3_ discoligamentous injuries, the two cases of anterior TD fracture without C_2‐3_ posterior displacement showed only C_2‐3_ ALL and disc injuries.

#### Other Associated Spine Injuries

All 14 TD fracture cases (100%) had at least two or more other associated C_2_ injuries, including in the pars interarticularis, pedicle, superior articular facet, transverse foramen, lamina, and spinous process. Among 14 patients with TD fracture of C_2_, 13 patients (92.9%) had typical or atypical hangman's fracture and all posterior TD fracture patients (100%) had hangman's fracture. Two TD fracture cases (14.3%) had associated C_1_ injuries such as posterior arch fracture. Four fracture cases (28.6%) had associated C_3‐7_ or thoracolumbar spine injuries such as spinous process or body fractures.

#### Surgical Treatment Methods and Fusion Status

**Fig. 1:**
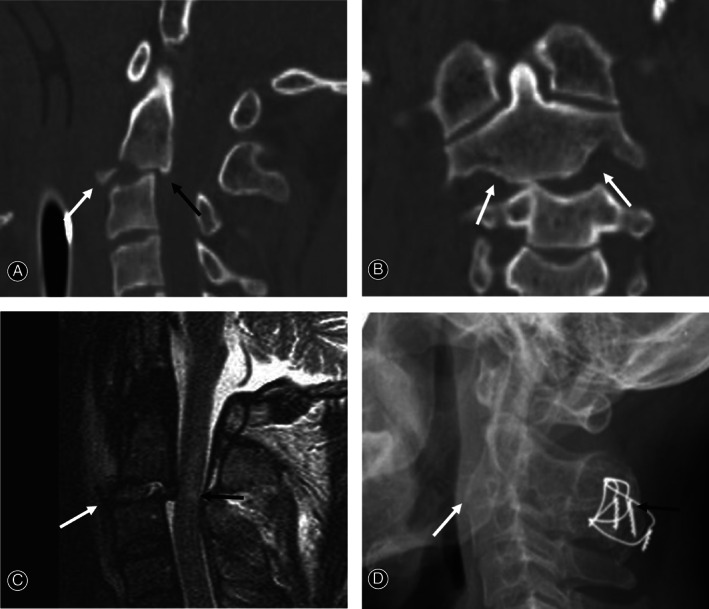
Sagittal computed tomography (CT) scan (A) showing anterior tear drop (TD) fracture (white arrow) and C_2‐3_ posterior slip (dark arrow) without hangman's fracture. Coronal CT scan (B) showing C_2‐3_ bilateral facet dislocation (white arrows). Sagittal magnetic resonance imaging (MRI) (C) showing C_2‐3_ discoligamentous injuries (white arrow) and spinal cord injury with intramedullary hemorrhage (dark arrow). At 24 months follow‐up after skull traction and surgery, lateral X‐ray (D) shows solid fusion of posterior C_2‐3_ fusion (dark arrow) and anterior TD fracture (white arrow).

**Fig. 2:**
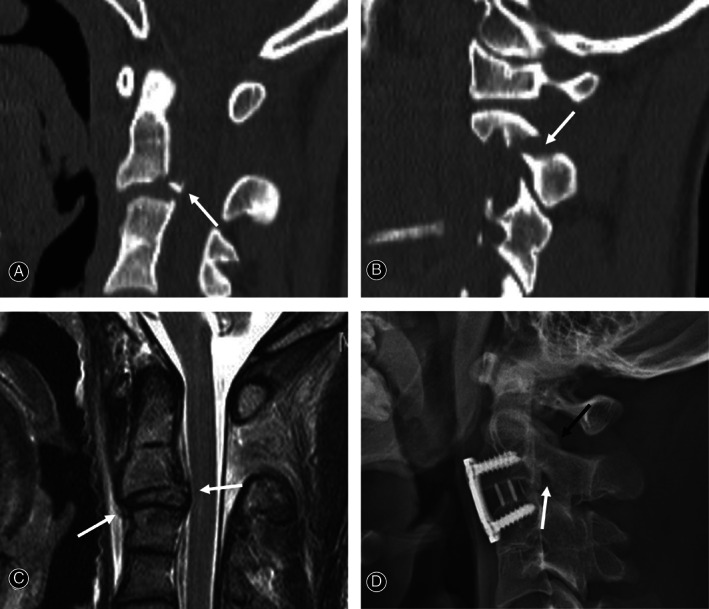
Sagittal and parasagittal computed tomography (CT) scans showing C_2‐3_ anterior slip and posterior tear drop (TD) fracture (white arrow) (A) and hangman's fracture (white arrow) (B). Sagittal magnetic resonance imaging (C) showing C_2‐3_ discoligamentous injuries (white arrows). At 44 months follow‐up after surgery, lateral X‐ray (D) showing solid fusion of C_2‐3_ anterior cervical discectomy and fusion and fractures of pedicle (white arrow) and lamina (dark arrow).

**Fig. 3:**
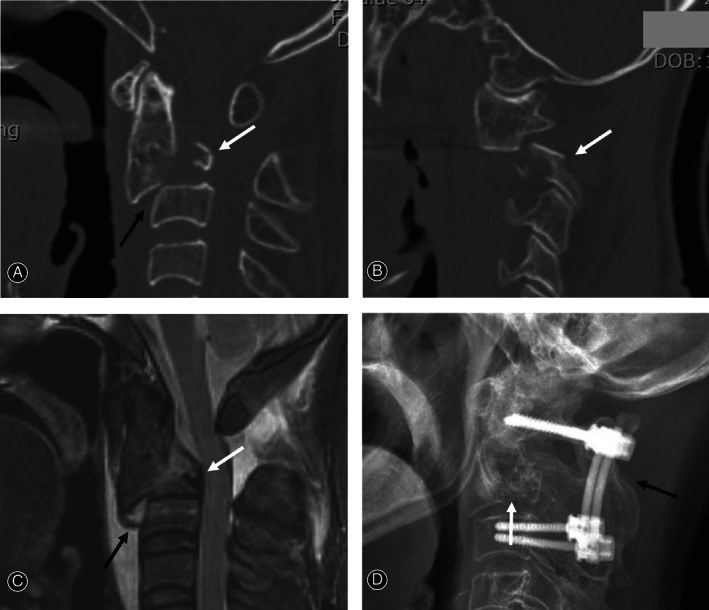
Sagittal and parasagittal computed tomography (CT) scans showing posterior tear drop (TD) fracture (white arrow) and C_2‐3_ anterior slip (dark arrow) (A) and hangman's fracture (white arrow) (B). Sagittal magnetic resonance imaging (MRI) (C) showing posterior TD fracture (white arrows) and complete C_2‐3_ discoligamentous injury including anterior longitudinal ligament, disc, and posterior longitudinal ligament at C_2‐3_ level (dark arrows). At 28 months follow‐up after surgery, lateral X‐ray (D) shows reduction of C_2‐3_ anterior slip and solid fusion of posterior C_1‐3_ fusion (dark arrow) and posterior TD fracture (white arrow).

Detailed analysis of 14 C_2_ TD fractures, including surgical treatment methods and fusion status, are summarized in Table 2. All four anterior TD fracture patients underwent posterior C_2‐3_ fusion using C_2_ pedicle screw and C_3_ lateral mass screw in two patients, C_2_ lamina screw and C_3_ lateral mass screw in one patient, and wiring in one patient (Fig. 1). While four posterior TD fracture patients underwent C_2‐3_ anterior cervical discectomy and fusion (ACDF) (Fig. 2), the remaining six underwent posterior C_2‐3_ fusion (five patients) or posterior C_1‐3_ fusion (one patient) (Fig. 3). For C_2‐3_ ACDF cases, a polyetheretherketone cage with autogenous cancellous iliac bone graft was used. For posterior C_2‐3_ or C_1‐3_ fusion, autogenous tricortical iliac bone graft was harvested and used. Based on hangman's fracture, 13 hangman's fracture patients underwent posterior fusion in nine patients and ACDF in four patients.

**TABLE 2:** Summary of surgically treated tear drop fractures of the C_2_

Patient		C_2‐3_ injury pattern		C_2‐3_ discoligamentous injury			Associated spine injury				Neurologic status*		Treatment outcomes	
No	Age/sex	Type of TD Fx	Direction of C_2‐3_ displacement	ALL	Disc	PLL	C_2_ axis	Hangman's fracture	C_1_ Atlas	C_3‐7_ or T/L spine	Admission	Follow‐up	Operation	Fusion
1	49/M	Anterior	Posterior	Yes	Yes	Yes	Body posterior vertical Fx Both pars Fx, Both TF Fx, SP Fx	Typical	n/a	C_3_ SP Fx	E	E	Posterior C_2‐3_ fusion w/ C_2_ PS and C_3_ LMS	Yes
2	37/M	Anterior	No	Yes	Yes	No	Body coronal and post oblique Fx Rt pedicle Fx, Rt pars Fx Rt SAF Fx, Lt lamina Fx	Atypical	Both post arch Fx	n/a	E	E	Posterior C_2‐3_ fusion w/ C_2_ PS and C_3_ LMS	Yes
3	50/F	Anterior	No	Yes	Yes	No	Body posterior vertical Fx Both pedicle Fx, Both pars Fx Both SAF Fx	Typical	n/a	C_6_ SP Fx	E	E	Posterior C_2‐3_ fusion w/ C_2_ LAS and C_3_ LMS	Yes
4	50/M	Anterior	Posterior	Yes	Yes	Yes	C_2‐3_ bilateral facet dislocation SCI, SP Fx	n/a	n/a	n/a	B	D	Posterior C_2‐3_ fusion w/ wiring	Yes
5	74/F	Posterior	Anterior	Yes	Yes	Yes	Both pedicle Fx, Both pars Fx Both SAF Fx	Typical	n/a	n/a	E	E	Posterior C_1‐3_ fusion w/ C_1_ LMS and C_3_ PS	Yes
6	37/F	Posterior	Anterior	Yes	Yes	Yes	Rt pedicle Fx, Lt pars Fx Rt SAF Fx, Rt TF Fx Lt lamina Fx	Atypical	n/a	n/a	E	E	Posterior C_2‐3_ fusion w/ C_2_ PS and C_3_ LMS	Yes
7	67/M	Posterior	Anterior	Yes	Yes	Yes	Both pars Fx, Lt SAF Fx, Both TF Fx	Typical	n/a	C_3_ coronal split Fx	E	E	Posterior C_2‐4_ fusion w/ C_2_ PS, C_3_ and C_4_ LMS	Yes
8	30/M	Posterior	Anterior	Yes	Yes	Yes	Lt pedicle Fx Lt SAF Fx, Lt TF Fx Rt lamina Fx	Atypical	n/a	n/a	E	E	Posterior C_2‐3_ fusion w/ C_2_ PS and C_3_ LMS	Yes
9	23/M	Posterior	Anterior	Yes	Yes	Yes	Body coronal Fx Both pars Fx, Both SAF Fx, Both TF Fx	Typical	n/a	T_7,8,9,10,11_ SP Fx	E	E	Posterior C_2‐3_ fusion w/ C_2_ PS and C_3_ LMS	Yes
10	43/F	Posterior	Anterior	Yes	Yes	Yes	Both pedicle Fx, Both pars Fx Both SAF Fx, Both TF Fx	Typical	Rt post arch Fx	n/a	E	E	ACDF C_2‐3_	Yes
11	19/M	Posterior	Anterior	Yes	Yes	Yes	Body coronal oblique Fx Lt pedicle Fx, Lt pars Fx Lt SAF Fx, Lt TF Fx	Atypical	n/a	n/a	E	E	ACDF C_2‐3_	Yes
12	64/F	Posterior	Anterior	Yes	Yes	Yes	Rt pedicle Fx, Rt pars Fx Lt SAF Fx, Lt TF Fx	Atypical	n/a	n/a	E	E	Posterior C_2‐3_ fusion w/ C_2_ PS and C_3_ LMS	Yes
13	59/M	Posterior	Anterior	Yes	Yes	Yes	Body coronal oblique Fx Both pedicle Fx, Both pars Fx Rt SAF Fx, Both TF Fx	Typical	n/a	n/a	E	E	ACDF C_2‐3_	Yes
14	35/F	Posterior	Anterior	Yes	Yes	Yes	Body coronal oblique Fx Lt pedicle Fx, Rt pars Fx Lt SAF Fx, Lt TF Fx Rt lamina Fx	Atypical	n/a	n/a	E	E	ACDF C_2‐3_	Yes

* Neurologic status is evaluated by ASIA impairment scale; ACDF, anterior cervical discectomy and fusion; ALL, anterior longitudinal ligament; ASIA indicates American Spinal Injury Association; Fx, fracture; LAS, lamina screw; LMS, lateral mass screw; n/a, not applicable; PLL, posterior longitudinal ligament; PS, pedicle screw; SAF, superior articular facet; SCI, spinal cord injury; SP, spinous process; TD, tear drop; TF, transverse foramen; T/L, thoracolumbar.

After surgery, all patients wore a Philadelphia brace for 12 weeks. At the last follow‐up after surgery, all 14 patients achieved solid fusion of the TD fracture, ACDF or posterior bone grafts, and associated C_2_ vertebral injuries including hangman's fracture.

### Clinical Results of C_2_ Tear Drop Fractures

#### Neurologic Status by American Spinal Injury Association (ASIA) Impairment Scale

At admission, 13 patients (92.9%) were neurologically intact, but the remaining patient (7.1%) had incomplete SCI. One anterior TD fracture with C_2‐3_ bilateral facet dislocation and posterior displacement sustained ASIA impairment scale grade B; however, SCI of ASIA impairment scale grade B was significantly improved to grade D after surgery.

#### Visual Analog Scale (VAS) for Neck Pain and Complications

The VAS rating for neck pain was significantly improved in all cases (5.9 ± 0.9 *vs* 2.2 ± 0.4, *P* < 0.001). No complications related to surgery, including death, surgical site infection, or iatrogenic neurologic deficit, occurred.

## Discussion

### Injury Mechanism of C_2_ Tear Drop Fractures

In general, extension TD fractures of the cervical spine are caused by hyperextension based on injury mechanism by Allen's classification^1^, ^2^, ^3^, ^4^, ^7^, ^8^, ^9^ and occur more commonly at C_2_ or C_3_. Hyperextension injury is the component of force most likely to cause anterior TD fracture of C_2_ ^19^, ^20^, ^21^. It is believed that our four anterior C_2_ TD fractures with or without C_2‐3_ posterior displacement (cases 1–4) were caused by hyperextension injury, as commonly mentioned in previous studies. In case 4, considering accompanying C_2‐3_ bilateral facet dislocation, it is thought that distraction force additionally acted on the hyperextension injury. In cases of posterior TD fractures (cases 5–14), considering associated C_2_ injuries of posterior bony elements, C_2‐3_ anterior displacement, C_2‐3_ kyphotic angulation, and C_2‐3_ discoligamentous injuries, the injury mechanism is thought to be a hyperextension compression injury followed by additional flexion force. In other words, it is thought that posterior TD fractures occur by a combination of hyperextension and compression forces, followed by a subsequent flexion force that causes anterior displacement and kyphotic angulation.

### Other Associated Spine Injuries Including Hangman's Fracture and C_2‐3_ Discoligamentous Injury

To date, no studies have analyzed the correlation between TD fractures of the C_2_ and other associated spine injuries. Tear drop fractures of the C_2_ can occur alone or in conjunction with other associated spine injuries, especially C_2‐3_ and other C_2_ injuries. Since associated C_2_ and C_2‐3_ injuries have a significant effect on treatment methods and outcomes, it is important to ensure that comprehensive injury analyses are performed to obtain satisfactory treatment outcomes^19^, ^20^, ^21^. Regarding the associated spine injuries, all 14 TD fracture cases had at least two or more other associated C_2_ injuries, including in the pars interarticularis, pedicle, superior articular facet, transverse foramen, lamina, and spinous process. Two TD fractures had C_1_ posterior arch fracture. Three TD fractures had spinous process fractures of C_3_, C_6_, and mid‐dorsal spine. One posterior TD fracture had coronal split fracture of C_3_ body. Such kinds of associated spine injuries strongly support hyperextension as the key contributing force to anterior TD fracture and the initial stage of posterior TD fracture.

Among 14 patients with TD fracture of C_2_, 13 patients (92.9%) had hangman's fracture. Among 13 hangman's fracture patients, seven patients (53.8%) had typical hangman's fracture and six patients (46.2%) had atypical hangman's fracture. All posterior TD fracture patients had hangman's fracture. Therefore, it is clinically important that although C_2_ TD fractures requiring surgery occurred together with many other associated C_2_ injuries, detailed analysis showed that most of them appear in the form of hangman's fractures.

In terms of C_2‐3_ discoligamentous injury, all 10 cases of posterior TD fracture with anterior C_2‐3_ displacement had C_2‐3_ discoligamentous injuries, including ALL, disc, and PLL. While two cases of anterior TD fracture with C_2‐3_ posterior displacement showed all three types of C_2‐3_ discoligamentous injuries, the two cases of anterior TD fracture without C_2‐3_ posterior displacement showed only ALL and disc injuries. Regardless of type of TD fracture, presence and extent of C_2‐3_ displacement are important factors determining the extent of C_2‐3_ discoligamentous injuries.

### Treatment Strategy of C_2_ Tear Drop Fractures

Previous studies with mostly small case numbers have reported satisfactory outcomes for anterior C_2_ TD fractures using conservative treatments^4^, ^7^, ^8^. Currently, conservative treatment is being used as a standardized treatment for anterior C_2_ TD fractures. However, detailed analyses of the previous studies revealed that the anterior C_2_ TD fractures that were successfully managed with conservative treatment were simple or small‐sized TD fractures. On the other hand, a few studies have reported that huge or large‐sized anterior TD fractures of C_2_ need to be treated surgically^12^, ^13^, ^14^, ^15^, ^16^, ^17^. However, the criteria for determining the indications and surgical approaches for surgery of C_2_ TD fractures have not been established and are controversial.

Associated C_2‐3_ displacement and discoligamentous injuries were identified in our all 14 TD fractures. If conservative treatment is performed, C_2‐3_ displacement is not well‐reduced, and the risk of failure is high. Since C_2‐3_ discoligamentous injuries cannot be cured by conservative treatment, they are likely to worsen C_2‐3_ instability and show poor results of conservative treatment. When deciding upon treatment methods in this study, spine surgeons judged that conservative treatment was insufficient for complex TD fractures of C_2_. As a result, all 14 TD fractures were treated surgically, and satisfactory radiological and clinical outcomes were achieved. All 14 patients achieved solid fusion of the TD fracture, anterior or posterior fusion, and associated C_2_ vertebral injuries including hangman's fracture and had a significant improvement in neck VAS. Therefore, the authors propose surgery as an appropriate treatment for anterior and posterior TD fractures of C_2_ with many comorbid C_2_ fractures and C_2‐3_ injuries including hangman's fracture and discoligamentous injury.

In terms of surgical treatment for C_2_ TD fractures, anterior, posterior, or combined surgeries can be performed. Each of the three surgical approaches has advantages and disadvantages^12^, ^13^, ^14^, ^15^, ^16^, ^17^, ^21^, ^22^. Therefore, spine surgeons need to decide upon appropriate surgical approaches for each patient by considering several factors: location of TD fracture, direction of C_2‐3_ displacement, C_2‐3_ discoligamentous injury, associated spine injuries including hangman's fracture, and neurologic deficit. In our study, all four anterior TD fracture and six out of 10 posterior TD fracture patients underwent posterior fusion. The remaining four posterior TD fracture patients underwent ACDF in consideration of other associated C_2_ fracture such as pedicle fracture or pars interarticularis fracture.

### Limitation and Strength of Current Study

There are two limitations in this study. First, since most of the TD fractures can be successfully managed with conservative treatments, surgically managed TD fractures of the C_2_ are very rare. Therefore, this study only included a small number of cases in spite of the collection of patients from four national trauma centers. Second, like other retrospective multicenter studies, we could not completely exclude potential errors of data collection. However, we believe that this is the first study to report successful surgical treatment outcomes of TD fractures of the C_2_ and to stratify C_2_ TD fractures into anterior and posterior TD fractures. We suggest that further studies with more cases are needed to investigate surgical treatment strategies suitable for anterior and posterior TD fracture of C_2_.

### Conclusions

Surgically managed TD fractures of the C_2_ showed a high incidence of other associated C_2_ and C_2‐3_ injuries including hangman's fracture and discoligamentous injury. Therefore, special attention and careful radiologic evaluation are needed to investigate the presence of associated C_2_ and C_2‐3_ injuries such as hangman's fracture and discoligamentous injury, which are likely to require surgery, when considering treatment options for anterior and posterior TD fractures of the C_2_.
